# Modeling of the SV40 DNA Replication Machine

**DOI:** 10.3390/genes3040742

**Published:** 2012-11-09

**Authors:** Daniel T Simmons

**Affiliations:** Department of Biological Sciences, University of Delaware, Newark, DE 19716, USA; E-Mail: dsimmons@udel.edu

**Keywords:** Simian Virus 40, DNA replication, T antigen, topoisomerase I, replication protein A, DNA polymerase alpha/primase, helicase, origin DNA unwinding

## Abstract

The mechanism of SV40 DNA replication is certainly not completely understood. The proteins that are necessary for replication have been known for quite some time, but how they work together to form a nanomachine capable of faithfully replicating the virus DNA is only partially understood. Some of the proteins involved have been crystallized and their 3D structures determined, and several EM reconstructions of SV40 T antigen have been generated. In addition, there is a fair amount of biochemical data that pinpoints the sites of interaction between various proteins. With this information, various models were assembled that show how the SV40 DNA replication nanomachine could be structured in three dimensional space. This process was aided by the use of a 3D docking program as well as fitting of structures. The advantage of the availability of these models is that they are experimentally testable and they provide an insight into how the replication machine could work. Another advantage is that it is possible to quickly compare newly published structures to the models in order to come up with improved models.

## Abbreviations

OBD: origin binding domain
DH: double hexamer
pol/prim: polymerase alpha/primase
RPA: replication protein A
ssDNA: single-stranded DNA
dsDNA: double-stranded DNA

## 1. Introduction

It has been clearly documented that the SV40 large T antigen forms a structure that acts as a scaffold for the construction of the viral replication factory [1,2,3]. This virally encoded protein forms a double hexamer structure over the virus origin of replication [4]. In the presence of ATP, the double hexamer bidirectionally unwinds the viral DNA [5,6]. Much effort has gone into how this structure forms and how it acts as a sequence dependent unwinding machine (for reviews, see [2,7]). The salient features are that the DNA binding domains of individual monomers recognize and bind to each of the four GAGGC pentanucleotide sequences that make up the center of the origin and in this way nucleate the formation of a double hexamer. The helicase domains are used to make contacts with the flanking origin sequences (the EP region and AT tract) [8] and, through subunit contacts, drive the formation of two hexameric helicases that contain a central channel through which DNA flows. This structure can form by itself over DNA. 

Replication of the viral DNA is, however, dependent on a number of cellular proteins that are recruited to this unwinding machine. Three cellular proteins, replication protein A (RPA), topoisomerase I (topo I) and DNA polymerase alpha/primase (pol/prim) are needed for maximal replication initiation [1,2,3]. At least three other proteins, polymerase delta, PCNA and replication factor C (RFC) are required for elongation synthesis [1,2,3]. There are published structures of various regions of T antigen, RPA and topo I. There is also information about the sites of interactions between T antigen and RPA, T antigen and pol/prim, T antigen and topo I, and topo I and RPA. It was the focus of this paper to develop models of how the DH functions as a helicase and how the ensuing replication factory could form and work.

## 2. Experimental Section

**Docking program:** The Z dock program (http://zlab.bu.edu) was used extensively in the construction of T antigen:topo I, T antigen:RPA, topo I:RPA and T antigen:topo I:RPA costructures.

**3D modeling program used:** All of the modeling and fittings were performed with the Swiss-pdb viewer program.

## 3. Results and Discussion

### 3.1. The Origin Binding Domain Double Hexamer

Since the origin binding domains of T antigen initiate the formation of the DH and since they are thought to attach in a head to head fashion in the center of the DH, they were a good starting point for the construction of the replication models. Various 3D structures of the origin binding domain have been determined [9,10,11,12]. There are two different dimer structures without DNA, two very similar non-interacting dimer structures in the presence of origin DNA and a reconstructed hexamer. The latter one, in particular, is believed to represent the structure of the OBDs at the center of the DH [9]. Importantly, some of the amino acids thought to participate in hexamer-hexamer interactions are known [13,14]. Analysis of the effect of mutations at these sites demonstrates that efficient circular DNA unwinding is dependent on contacts between hexamers. 

**Figure 1:**
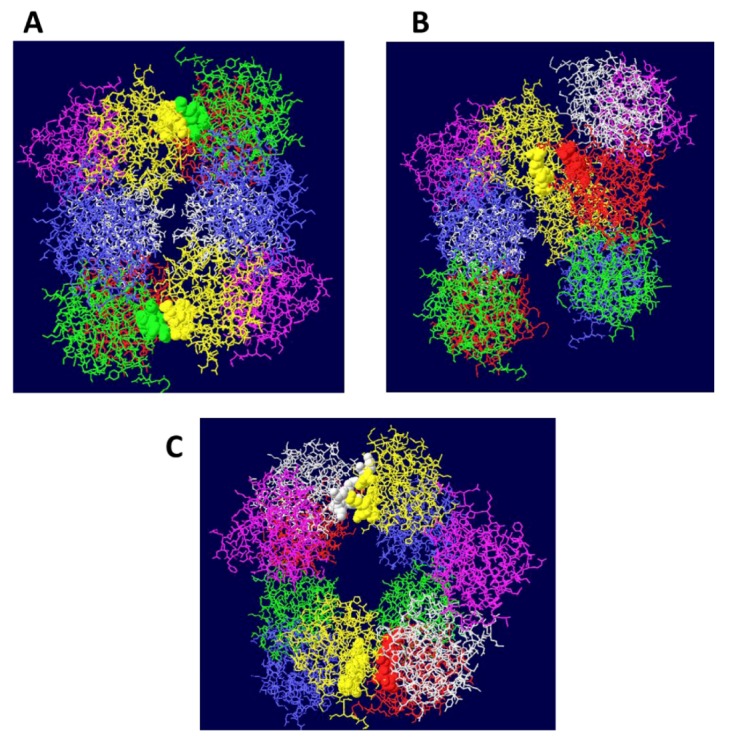
Side views of three different models of OBD hexamer-hexamer stacking. In (**A**), the “outlying” OBD monomers (in purple) are on opposite faces of the DH and the two hexamers have been brought in as close as possible to maximize the contact surfaces. In (**B**), the contacting residues are those in region B2 of the OBD and correspond to residues 216-220. In (**C**) the “outlying” OBD monomers are directly opposite one another.

**Figure 2:**
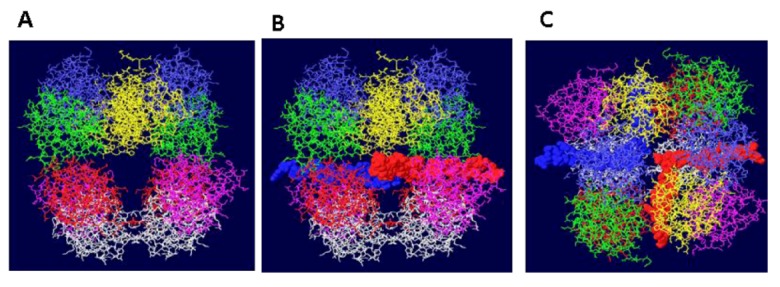
Threading of SSDNA through the OBD DH. **(A**) The structure shown in 1A has been rotated by 90 degrees to show the two side holes of this DH structure. In (**B**), the same representation is shown with single stranded DNA. One strand (red) goes through the central channel of the OBD hexamer on the right and out through the side hole towards the viewer. The other strand (blue) goes through the other OBD hexamer and exits towards the back. (**C**) The structure shown in 2B has been rotated by 90 degrees to show the DNA emerging at the bottom and top.

**Figure 3:**
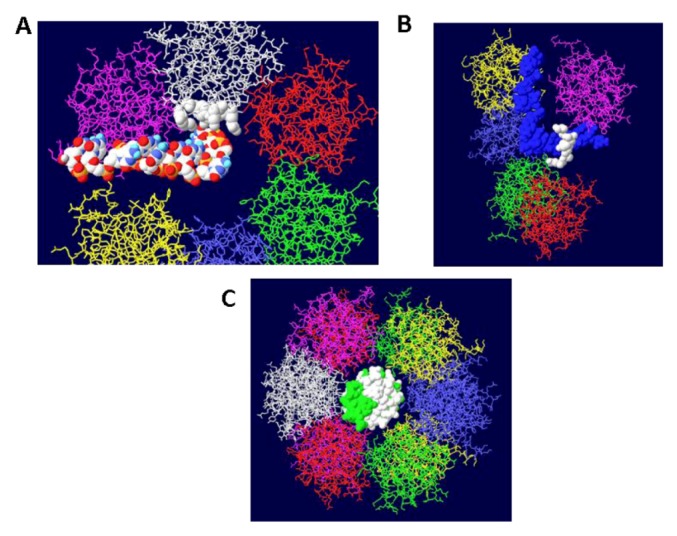
DNA in the channel of the OBD hexamers. (**A**) A single OBD hexamer is shown with its central channel in the middle. Single stranded DNA is shown to transit into the central channel, pass by four critical residues for DNA unwinding (white) and is threaded through the gap generated by the two terminal OBD monomers (purple and yellow). (**B**) A side view of a similar structure showing single stranded DNA passing within a trough generated by the four residues. (**C**) Face-on view of an OBD DH with double stranded DNA going through the central channel.

The OBD hexamer structure is an open ring (lock washer) where the terminal OBD monomers are separated by a gap that is large enough to accommodate at least ssDNA [9]. However, there is no DNA in the structure and so it is not known for certain that DNA flows through that gap but NMR analyses of the OBD associated with single stranded DNA support this idea [15]. In addition, mutagenesis of some of the residues located close to the gap shows that they are essential for DNA replication [16]. The first modeling attempt was to come up with different ways to join two OBD hexamers head to head consistent with the mutagenesis data. Not surprisingly, it turns out that several OBD DH structures are possible. The three structures with the greatest number of contact points between OBD hexamers were considered further (Figure 1). Evaluation of these possible structures is fortunately aided by recent cryo EM reconstruction studies of DHs [17,18,19,20,21]. Structure B in Figure 1 has the two OBD hexamers offset relative to one another but this is not consistent with the EM images that show near perfect alignment of the two OBD hexamers. Further, there is no obvious side holes that are seen in the EM images and that are hypothesized to serve as entry/exit sites for ssDNA [21]. Structure C shows the two “outlying” OBD monomers in each hexamer (purple monomers) opposite one another. This generates an OBD DH structure that has less extensive contacts between hexamers, and although not altogether bad, a head to head arrangement suggests symmetry around a central point as opposed to a plane (mirror images). The major problem with structure C is that it would mean that the helicase domains are skewed relative to one another because the outlying OBD monomers are on the same side. A skewed arrangement of helicase domains is seen in the presence of ATP [21] but not in the absence of hydrolyzable ATP, suggesting a more symmetrical arrangement of the OBD hexamers. Only structure A appears to be consistent with most of the data. It predicts hexamer to hexamer contact sites that are known (residues 216, 217 and 218) as well others that are not (residues 167 and 245 through 249). Interestingly, the latter set of residues, like the first, cannot be mutated without affecting DNA replication activity [13,22,23]. The structure has the appealing qualities that extensive surface interactions exist between the two hexamers, that the structure is symmetrical, and that two side holes are formed (Figure 2A). Modeling demonstrates that ssDNA could easily pass through these holes (Figure 2B,C). The additional attractive quality of this model is that the ssDNA could pass very close to four residues that are thought to participate in the threading of the ssDNA during DNA unwinding [16] (Figure 3A). Mutations of these residues reduce circular DNA unwinding and DNA replication activities. Importantly, the defects can be rescued by the addition of the single-stranded DNA binding protein RPA and/or with pol/prim indicating that the role of the residues is in guiding the single stranded DNA out of the structure or perhaps acting to prevent reverse movement of the DNA [16]. The four residues form a trough that can accommodate ssDNA (Figure 3B) (also see Figure 10 in reference [16]). In the model, the DNA bends very close to the trough and then exits through the side channel. Prior to unwinding, the double stranded DNA can easily fit within the central channel of the OBD DH (Figure 3C).

### 3.2. Structure of theDouble Hexamer

**Figure 4:**
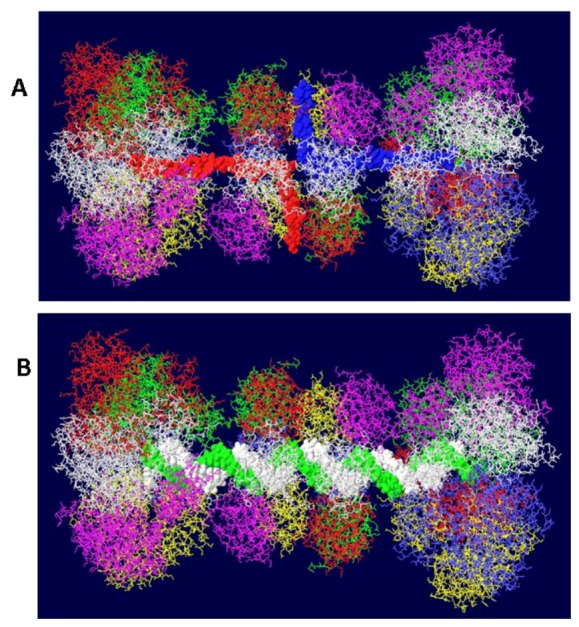
Views of DHs with single and double stranded DNA. Two helicase domains were positioned close to the OBD hexamers to create a model of the T antigen DH viewed from the side. (**A**) Single stranded DNA is shown threading through part of each helicase domain and through the OBD hexamers emerging at the top and bottom. (**B**) Double stranded DNA occupying the central channel of the DH. Only the first helicase subdomains (the small tiers) are filled with DNA. The large tier helicase domains are at the ends of the structure.

Structure A was used as a starting point for modeling the complete DH. The helicase domain hexamer structure has been determined in various forms [24,25]. These forms include a nucleotide-free structure [24], one containing ADP and one with ATP [25]. The ADP form was used for modeling ssDNA through the helicase in a later step, but for the initial modeling of the DH, the nucleotide-free form was used, primarily because it has the largest central channel. There are other small differences between these structures. As with OBD hexamer to hexamer contacts, there are many ways to join the OBD and helicase hexamer together. Seven amino acids in between these two domains are missing in the structures and it is therefore impossible to guess exactly how they fit. Nevertheless, Figure 4A shows a model where the C-terminus of the OBD and the N-terminus of the helicase domain are reasonably close to one another. In addition, the “outlying” OBD monomers are positioned in between two helicase domains so as to bring the structures as close to one another as possible. The cryo EM images [17,18,19,20,21] clearly show separate OBD and helicase domains and they appear to be pretty close to one another. The central channel made by the large tiers of the helicase domains in the nucleotide-free state is not quite large enough to easily accommodate dsDNA, but the small tiers do make a sizable central channel. It is therefore possible that dsDNA could be accommodated through most of the DH central channel (Figure 4B). Since the central channels of the ADP bound and ATP bound forms of the helicase domains are smaller, these structures will most likely not permit dsDNA within the large tier subdomains.

How, then, do the large tiers of the helicase domains engage DNA? There are two main schools of thought. One is that dsDNA first fits into the entire structure (the central channel would have to expand) and upon DNA melting and unwinding, one strand gets displaced to the outside of the helicase domain and the central channel of the large tier subdomains closes on the other strand [24,25,26]. How this might happen is of considerable interest. The second idea is that the DNA is melted as each hexamer is assembled from individual monomers permitting only ssDNA to occupy the central channel of the helicase domain [27]. There are data supporting each view; however, the most telling piece of information is that DHs do form in the presence of ADP or non hydrolyzable ATP analogue and that in this structure, the DNA is partially melted only in the EP region and not melted but only slightly distorted in the AT tract [28]. These two regions of the DNA coincide with the positions of the helicase large tiers [8]. It is therefore almost certain that dsDNA would occupy most of the central channel initially except in the large tiers, as shown in Figure 4B. Even for the AT tract region, it is likely that the undenatured DNA occupies the channel in the large tier subdomains because this region of the origin is untwisted and therefore has a smaller diameter. After melting of the origin, ssDNA would occupy all of the central channels and pass through the side holes in the OBD DH (Figure 4A). So, where is the DNA within the large tiers initially?

### 3.3. Mechanism of DNA Unwinding

**Figure 5:**
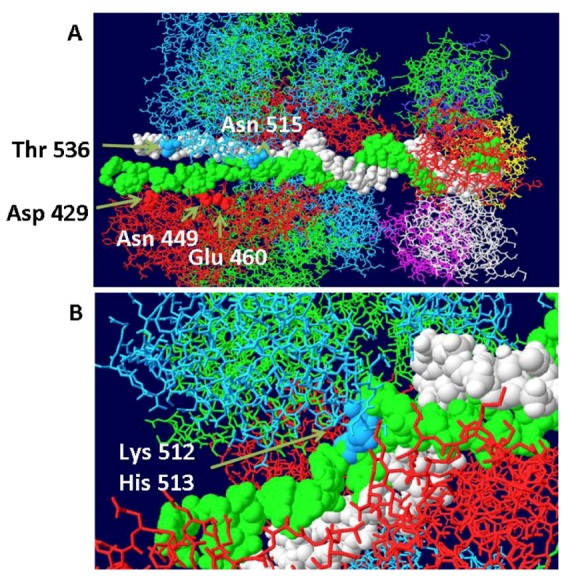
Threading of DNA through the large tier subdomains. Double stranded DNA is shown going through the central channel of the small tier subdomains and separate in the large tiers. One strand (white) continues within the central channel of the large tier subdomains. The other (green) is threaded within a hydrophilic channel that lies between monomer helicase domains (red and blue subunits). As shown in (**A**), it passes close to several residues that have been shown to be important in origin unwinding. (**B**) Close-up of same structure showing the location of residues 512 and 513 in the β-hairpin of the blue subunit.

Examination of the structure of the hexameric helicase clearly shows the existence of curved channels formed by neighboring large tier subdomains [29]. Each of the six channels is lined with many hydrophilic or charged residues and has therefore been called the hydrophilic channel. Those channels are distinct from the central channel except at the center where the two converge. Extensive mutagenesis of the residues that line the hydrophilic channels clearly demonstrates that these regions are essential for origin DNA unwinding [29]. Most mutants were completely defective in unwinding origin containing dsDNA but not in helicase activity. Based on extensive biochemical assays, it was determined that at least one of the hydrophilic channels is required for positioning the ssDNA formed after unwinding of the origin. The most reasonable interpretation of all the data is that one of the hydrophilic channels is used to transit one strand of DNA to the outside as the hexamer assembles from individual monomers while the other strand becomes positioned within the central channel. It is possible to model ssDNA in the hydrophilic channel and come close to all of the main residues identified in that study. This model is shown in Figure 5A,B. It basically hypothesizes that as each hexamer forms, the DNA in the EP region, at least, is melted and that one strand associates with various residues in the hydrophilic channel as shown in the figure. The DNA strand in the hydrophilic channel is then moved between helicase trimers to the outside of the hexameric helicase where it would function as the template strand for DNA replication. In all of the three published helicase structures, there is insufficient room in the hydrophilic channels to accommodate ssDNA. To come up with this model, two trimers of the ADP bound structures [25] were positioned sufficiently apart so that ssDNA could pass between them. The ADP-bound form was chosen because it has the widest hydrophilic channels. Trimers were selected because there is some evidence that they represent an intermediate in the formation of hexamers, at least with the related papilloma virus E1 helicase [30]. The model was also built to show that the junction between DS and SS DNA is at the beta hairpin residues (Lys512 and His513) that have been shown to be critical to the binding of ssDNA during helicase action [24,25]. An equivalent beta hairpin structure exists within the papilloma virus E1 helicase [31]. Presumably, after one strand becomes associated with the hydrophilic channel as shown, it is pushed to the outside of the helicase and the two trimers close resulting in a hexamer with the other DNA strand imbedded in its central channel as described by Enemark [31].

### 3.4. RPA-DH Structure

**Figure 6:**
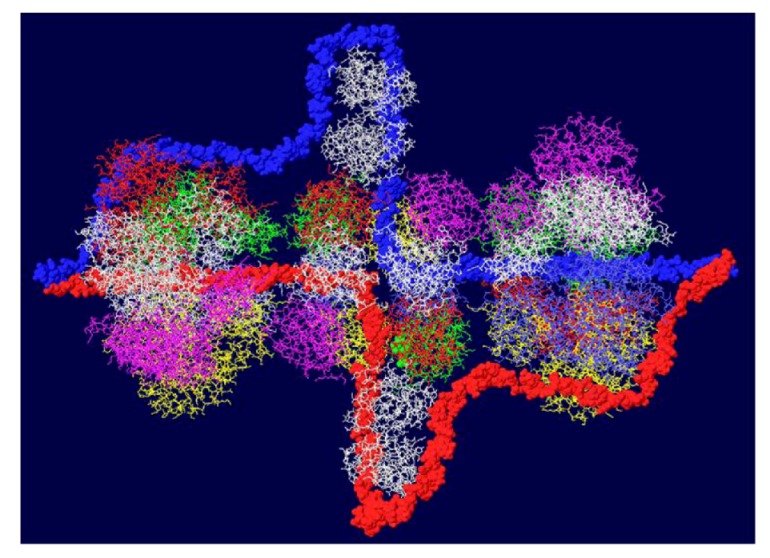
Complete model of single-stranded DNA threading in DH and associated RPA 70. Each DNA strand is shown going through the central channel of one hexamer, passing through a side hole of the OBD DH and going through RPA and around the other helicase domains. RPA is shown in white at the top and bottom.

Once the double hexamer structure was modeled with ssDNA going through the structure, the next step was to position RPA. This trimeric ssDNA binding protein has been crystallized only in parts. The structure of part of the largest subunit (RPA70) associated with ssDNA has been determined [32] as has the structure of the trimeric core of the protein [33]. RPA32 in association with an OBD monomer has been solved by NMR [34]. Unfortunately, none of these structures overlap so it is not possible to position the entire protein on the T antigen structure. Nevertheless, we do know that the RPA molecule should be positioned close to one of the OBD subunits [35] and we make the major assumption that the ssDNA that emerges from the center of the OBD DH passes through RPA70. In this way, the ssDNA would be grabbed by RPA from the DH and then presented to the DNA polymerase for the enzyme to copy. A complete model showing threaded ssDNA was built with RPA 70 (Figure 6). Each strand emerges from the center of the DH into RPA and wraps around the helicase domain of the other hexamer. Each external strand is then used as a template for DNA synthesis. 

### 3.5. Placement of Topoisomerase I at the C Terminal Regions

**Figure 7:**
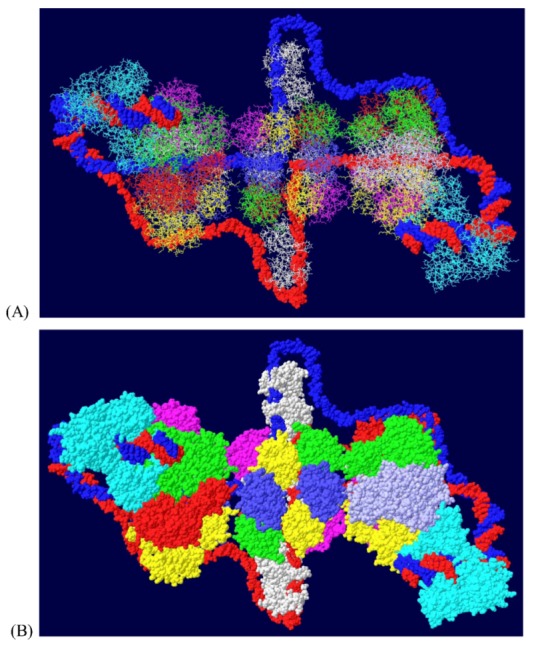
Complete model of DNA threading through DH with associated RPA and C-terminal bound topo I. Topo I (shown in cyan) was fitted on the back end of each helicase where it interacts with residues near the C-terminal end of T antigen. Double stranded DNA is shown going through topo I (present in original structure), bend, and separate into its two strands, one going through the central channel of the helicase and one going around. (**A**) Representation of the 3D structure with only the DNA shown space filled. (**B**) Space filled representation of entire model.

Another cellular component of the replication machine is topo I [1,2]. There are two binding sites on T antigen for topo I, near the C-terminal end and at the junction between the OBD and the J domain [36,37]. Of these, the C-terminal binding site has been better characterized but both seem to be involved in efficient DNA replication [38]. Extensive mutagenesis of the C-terminal binding site on T antigen has led to a model of the association of the two proteins for one another [38]. This model shows topo I bound to the back ends of the DH with its central hole, which contains dsDNA, facing in the same direction as the central channel of the DH. We had proposed from this, that dsDNA moves through topo I, bends about 165^o^ and separates into individual strands at the back ends of the DH. The topo I and associated DNA were added to our model with RPA (Figure 7A). A space filled rendition is shown in Figure 7B. This structure illustrates the tight association of the cellular proteins with the DH unwinding machine. 

### 3.6. Placement of Part of pol/prim on the RPA/Topo I/T DH

**Figure 8:**
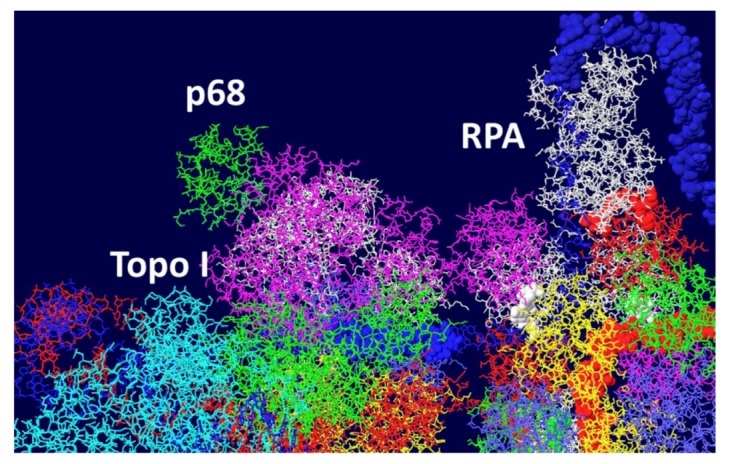
Model of the Topo I/RPA/T DH with part of pol/prim. A portion of the pol/prim p68 subunit was fitted onto the Topo I/RPA/T DH structure shown in Figure 7. Topo I is in cyan, p68 in green and RPA in white. The rest of the pol/prim molecule may be able to interact with topo I and RPA.

There is little structural information on pol/prim. This four subunit enzyme complex synthesizes the RNA primer using its p48 primase subunit and extends it with DNA using its p180 polymerase subunit [1,2]. Each of these two subunits would have to accommodate the passing ssDNA template in order to copy it. It has been known for some time that this enzyme complex associates with both RPA and T antigen [39,40,41]. The thinking is that pol/prim should be positioned over the helicase domain of T antigen in such a way that it extends to RPA in order to capture its bound ssDNA. Recently, a structure of the T antigen helicase domain associated with a portion of the p68 subunit has been determined by X-ray crystallography [42]. This pol/prim subunit has no catalytic activity but is required for efficient DNA replication [43]. The segment of the p68 subunit was fitted onto the RPA/Topo I/T DH structure choosing a T antigen subunit that would place p68 on the same face of the DH as RPA and close enough to the bound topo I to possibly interact with it as well. We demonstrated [44] that the major function of the C-terminal bound topo I is to stimulate pol/prim to make RNA/DNA primers. It is therefore hypothesized that pol/prim also interacts with topo I during initiation of new chains. Pol/prim is a large protein, larger than each hexameric helicase. So, although only a very small portion of pol/prim can be fitted onto the DH model (Figure 8), the remainder of the molecule might be able to interact with RPA and topo I.

### 3.7. Position of the J Domain and Topo I at N Terminal Regions

**Figure 9:**
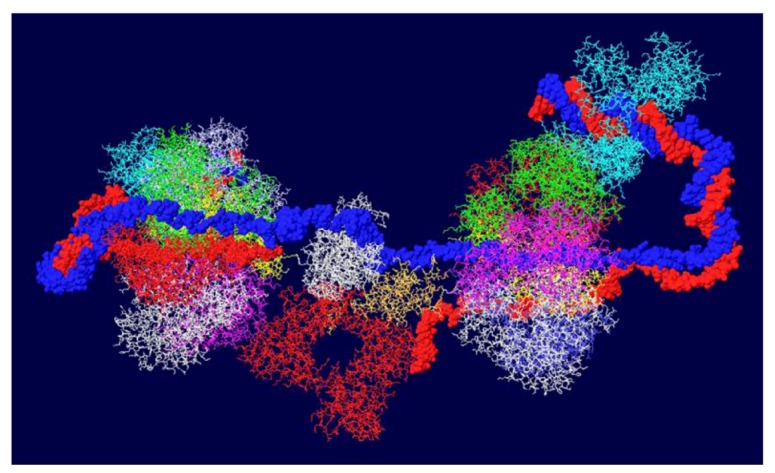
Model of DH with topo I bound to the N-terminal site. The structure shown in Figure 7 was fitted onto a model of topo I:J domain:RPA70 (Figure 10C). There are no clashes in this structure, but for clarity, the OBDs, part of the red DNA strand and its associated RPA were omitted. The model was also turned to show the molecule from the “top”; that is the blue DNA strand is shown to go through RPA and away from the viewer. The DNA from within the N-terminal bound topo I was also deleted.

Since topo I is also known to bind near the N-terminal end [36], this was modeled as well. Modeling of the second topo I enzyme was dependent on extensive docking program analyses. Topo I binds primarily to the linker region between the J domain and the OBD [37]. The structure of the J domain with its linker region has been determined in association with part of the Rb protein [45]. In order to model the topo I bound to the N-terminal region, Z-dock models were obtained for topo I-J domain costructures as well as for costructures of RPA and J. In addition to the binding of RPA to the OBD, there is evidence that RPA binds to part of the J domain (residues 89-97) [46]. Docking models were also obtained with all three proteins. The best model, as determined by consistency with the biochemistry data, was fitted onto the RPA-DH structure shown above. Fitting was performed by matching the structure of the RPA molecules in each model. This allowed us to position both the J domain and topo I on the surface of the OBD DH-RPA (Figure 9). The structure shown is from the “top”; that is, the blue strand threads through RPA and moves away from the viewer. For clarity, the red strand has been deleted in parts as have the two OBD hexamers. No DNA is shown within the topo I molecule because there’s no reason it would contain DNA. Notice that the J domain (in yellow) is wedged between the two cellular proteins. This concept is consistent with the importance of the J domain in DNA replication in vivo, presumably in order to properly interact with these two cellular proteins.

**Figure 10:**
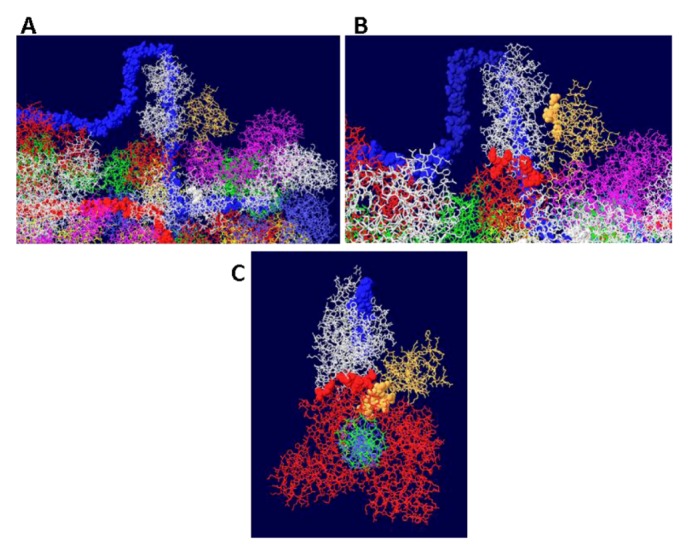
Details of T-RPA, J domain-topo I, and topo I-RPA interactions in the model. (**A**) the model in Figure 9 was rotated to illustrate the position of the J domain relative to an OBD monomer. The J domain (gold) lies on the surface of the purple OBD monomer. (**B**) Close-up of J domain-RPA and OBD-RPA interaction surfaces. The highlighted gold residues are those of the J domain contacting RPA and the highlighted red residues are those of the OBD monomer contacting RPA. (**C**) “Top” view of the J: RPA:topo I costructure model. This is approximately the same orientation as in Figure 9 but the double stranded DNA going through topo I is included here. The highlighted J domain gold residues are those that contact topo I and the highlighted red residues are those of topo I contacting RPA70.

The J domain is seen as a flat structure that covers the surface of the OBD hexamer in EM images [21]. Consistent with this, the J domain lies over the OBD in the model (Figure 10A). The interactions between T antigen and RPA are shown in Figure 10B. This figure shows that RPA is contacted by two sections of T antigen: the OBD and the J domain. The position of topo I in the complex (with DNA added for illustration) is shown in Figure 10C. Sites of interaction between topo I and RPA and topo I and the J domain are highlighted.

### 3.8. What Doesn’t Work

**Figure S1:**
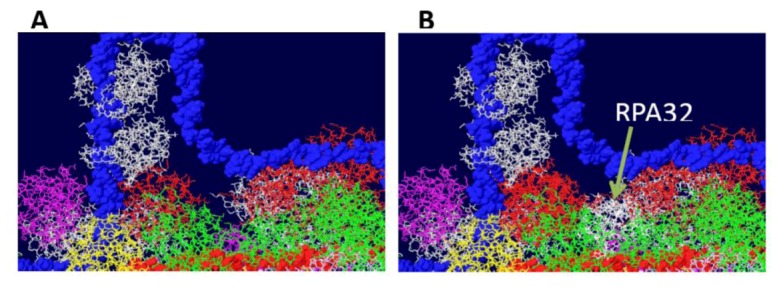
Fitting of the OBD:RPA32 structure on the DH. The OBD:RPA32 structure was fitted onto a DH by combining the OBD structure of the first with various OBD monomers of the second. A) image of DH surface and RPA70 before fitting. B) The red OBD monomer of the right hexamer was fitted with the OBD (also red) of the OBD:RPA32 costructure. The RPA32 molecule, shown in white, clashes with the helicase domain and is far removed from the RPA70 subunit.

**Figure S2:**
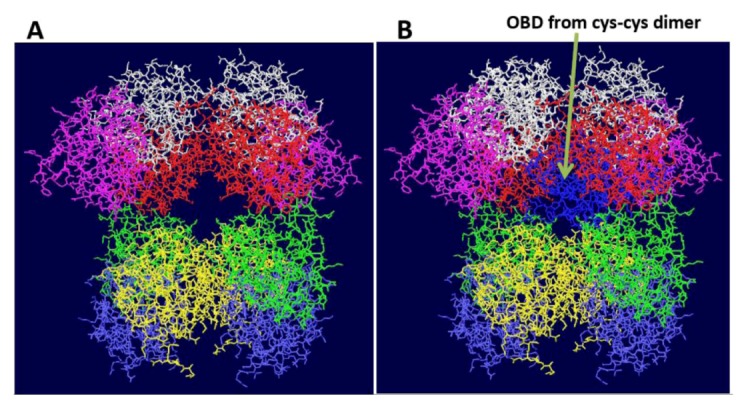
Fitting of OBD dimer structures on the DH. The cys-cys dimer OBD structure [9] was fitted onto one of the OBD monomers of the OBD DH shown in Figure 1A. (A) Starting structure of OBD DH. B) The white OBD monomer on the left was fitted on to one of the OBD monomers (also in white) of the cys-cys dimer. The other monomer of the dimer (blue) does not match any of the other OBDs. Similar problems were obtained with all of the model OBD double hexamers shown in Figure 1.

It was not possible to fit the OBD:RPA 32 costructure [34] onto an OBD DH without changing it greatly. Clashes occurred in all cases (Figure S1). Likewise, it was not possible to fit any of the OBD dimer structures onto the OBD DH. Some of the OBD dimer structures were predicted to show contacts between hexamers, but it was not possible to fit them into the DH structure. One example of this with the Cys-Cys dimer structure [8] is shown in Figure S2. It is possible that the OBD:RPA32 and OBD dimer structures participate in unknown intermediate steps during DNA replication and/or unwinding. 

## 4. Conclusions

Based on structural information and biochemical data, models were generated to show how a viral helicase could unwind DNA and act as framework for the assembly of the DNA replication machine. The models described here are testable. Mutations can be introduced at predicted sites of interaction to determine if they affect binding, DNA unwinding and replication. One of the lessons learned is the fact that it is possible to combine imaging data with mutagenesis to come up with plausible models. As more structural and biochemical data becomes available, more refined models can be quickly generated. Pdb files of any of the models described in this paper can be requested from the author at dsimmons@udel.edu.
